# Canine circovirus: emergence, adaptation, and challenges for animal and public health

**DOI:** 10.3389/fvets.2025.1535650

**Published:** 2025-03-28

**Authors:** Diego Ferreira da Silva, Milene Ciola, Verônica de Oliveira Lopes, Débora Rhayanne Medeiros Matias, Tarley Santos Oliveira, Alessandra Marnie Martins Gomes de Castro

**Affiliations:** ^1^School of Nursing, University of São Paulo (EEUSP), Graduate Program in Nursing in Adult Health (PROESA), São Paulo, Brazil; ^2^Paulista University (UNIP), Graduate Program in Environmental and Experimental Pathology, São Paulo, Brazil; ^3^Department of Undergraduate Studies in Veterinary Medicine, Anclivepa College, São Paulo, Brazil

**Keywords:** CanineCV, *Circoviridae*, *Porcine circovirus*, public health, emerging, pig disease

## Abstract

**Introduction:**

Canine circovirus (CanineCV) is an emerging pathogen with a significant impact on animal health and potential zoonotic risks. This study addresses its characterization, epidemiology, pathogenesis, and diagnostics, emphasizing its relevance within the One Health approach.

**Background:**

The increasing detection of CanineCV across various species and regions highlights its genetic adaptability and cross-species transmission potential. Furthermore, growing interactions among domestic animals, wildlife, and humans amplify the need to understand its public and animal health implications.

**Objective:**

To analyze the biology, epidemiology, and diagnostic challenges of CanineCV, focusing on its genetic evolution, interactions with co-infections, and implications for control strategies.

**Methods:**

A systematic literature review was conducted, synthesizing data from epidemiological, genomic, and clinical studies. Molecular techniques, such as PCR and qPCR, were evaluated for their efficacy in virus detection and quantification.

**Results:**

Canine circovirus exhibits high genetic variability and has been detected in diverse species and tissues. Co-infections, including parvovirus and adenovirus, exacerbate clinical signs, primarily gastrointestinal, and respiratory. Advances in diagnostics, such as real-time PCR and *in situ* hybridization, have demonstrated increased sensitivity in viral detection.

**Conclusion:**

Canine circovirus poses a growing challenge to animal health and a potential threat to public health due to its genetic plasticity and adaptability to multiple hosts. Continuous research is essential to understand its pathogenesis, develop effective control measures, and mitigate risks in diverse ecosystems.

## Introduction

Pets play a significant role in contemporary society, transcending their historical function as mere guardians or domestic helpers. According to the International Federation for Animal Health (IFAH), “pet” refers to any animal kept by humans for companionship, recreation, or as part of the family unit, encompassing not only dogs and cats but also birds, reptiles, fish, rodents, and other small mammals (2, 3). The human-animal relationship has evolved over the decades, shifting from a utilitarian interaction to an emotional bond, with documented benefits for the mental and physical health of their owners, such as stress reduction, increased social engagement, and improved quality of life (4). In Brazil, this scenario is widely reflected in households, where 47.9 million families own at least one pet, representing ~46.1% of all national households, according to the Brazilian Institute of Geography and Statistics [IBGE; (1)]. This figure becomes even more remarkable when considering that the country leads globally in the number of small dogs per capita and holds a prominent position in the ownership of cats and other animals. The broad definition of the term “pet” not only reflects the diversity of species that share domestic spaces with humans but also highlights the complexity of human-animal interactions, which are influenced by cultural, socioeconomic, and environmental factors (73). Thus, understanding the concept of “pet” in its entirety is essential to contextualizing health impacts, both human and animal, within the One Health approach (5–7).

Canine circovirus (CanineCV) is an emerging virus with a significant impact, particularly in the absence of vaccines. This virus often displays a variety of clinical signs, which can be further complicated by co-infections, potentially altering the clinical presentation. Additionally, the potential for zoonotic transmission cannot be excluded, as other viruses within the same genus, which includes human circovirus, are being considered for such transmission (8–10).

Canine circovirus belongs to the genus *Circovirus* within the family *Circoviridae* According to the classification of the International Committee on Taxonomy of Viruses (ICTV), species demarcation within the *Circoviridae* family is based on at least 80% nucleotide identity across the entire genome, along with structural and organizational characteristics. An essential criterion is the location of the replication origin (ori) relative to the coding regions. In members of the genus Circovirus, the ori is located on the same strand that encodes the replication-associated protein (Rep), while in the genus Cyclovirus, the ori is situated on the strand that encodes the capsid protein [Cap; (10, 11)]. Additionally, the genomes of these viruses exhibit an ambisense organization with two primary open reading frames (ORFs) responsible for encoding the Rep and Cap proteins, with replication occurring through a rolling circle replication mechanism.

The diversity within this family has been significantly expanded through metagenomic sequencing and degenerate PCR methods, revealing a broad distribution among mammals, birds, and even invertebrates. Phylogenetic studies indicate a closer relationship among circoviruses detected in mammals, whereas those found in birds and fish display greater genetic distance, reflecting their complex evolution. These criteria and technological advancements not only facilitate taxonomic classification but also provide a broader understanding of the biology and ecology of these viruses, which are essential for epidemiological and viral evolution studies (12, 13).

Canine circovirus was first identified in 2012 after the extraction of viral nucleic acid from a set of canine serum samples in the United States. In 2013, the complete genome was characterized in California (USA) and, after a year later, the virus was reported in a young dog in Italy. Since its identification, CanineCV has been associated with conditions such as vasculitis, hemorrhagic gastroenteritis, and diarrhea (57) and has been reported in all continents, except Oceania (14–19).

Canine circovirus, like other circovirus species, poses significant challenges in classification due to notable genetic variability. It is crucial to establish common terminology with robust classification criteria, ensuring reproducible results and promoting essential advancements in understanding diseases associated with the virus. This includes assessing the impact of coinfections on clinical signs to comprehend its effects on animal health and potential implications for public health.

## Viral characterization and diversity

Canine circovirus are non-enveloped icosahedral viruses with a single-stranded circular DNA genome of ~2 kb. As previously mentioned, they belong to the *Circoviridae* family. The classification of CanineCV as a new species within the circovirus genus occurred because, according to criteria set by the International Committee on Taxonomy of Viruses (10), circoviruses must share more than 75% nucleotide identity across their complete genome and more than 70% sequence identity in their capsid protein sequences to be considered the same species. Despite being genetically closer to porcine circovirus, in the study identifying the complete genome sequence of the first canine circovirus, the capsid (Cap) and replicase (Rep) proteins of CanineCV shared <25% and 50% identity, respectively, with circoviruses from other animals (8).

**Figure 1:**
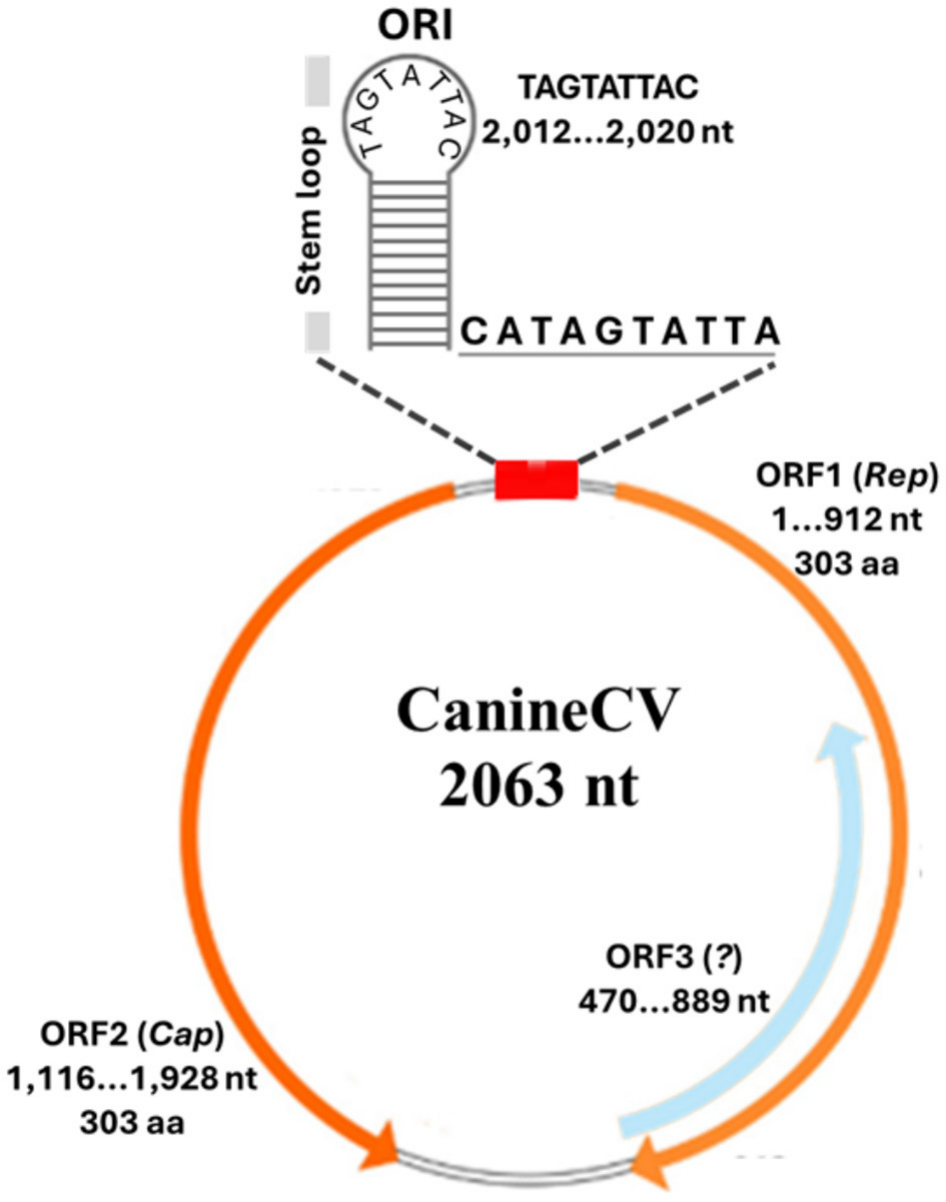
Schematic representation of the CanineCV genome. Information on open reading frames [ORF1, ORF2, ORF3; (8, 19, 20)].

The CanineCV genome is a circular single-stranded DNA with 2,063 nucleotides (nt) that comprises two open reading frames (ORFs) on complementary strands oriented in opposite directions. ORF1, with 911 nt, encodes the replicase protein (303 amino acids), which is essential for viral replication. ORF2, with 811 nt, encodes the capsid protein (270 amino acids), which has a structural function. The genome also contains two intergenic non-coding regions that are 135 and 203 nucleotides long. At the replication origin (TAG TAT TACA), there is a palindromic sequence of 12 nt pairs and a 10-nucleotide open loop (CAT AGT ATT A). The amino terminus of the proposed capsid protein features a 30-amino-acid arginine-rich region, like those found in other animal circoviruses. Additionally, a third ORF (ORF-3) was identified in the antisense strand of ORF-1 from a Thailand strain, although its function is still unknown [(8, 20); Figure 1].

**Figure 2:**
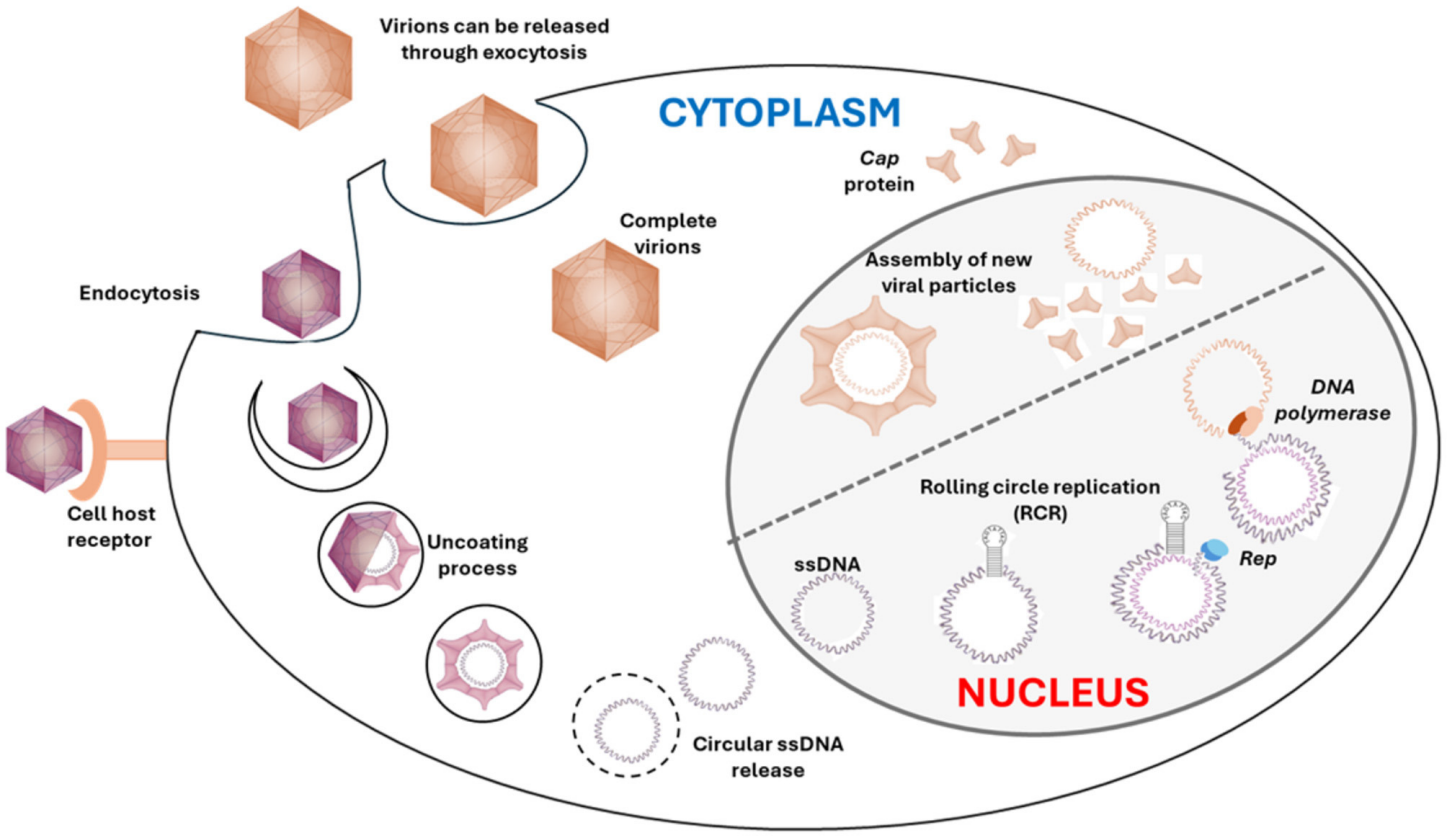
Schematic representation of the CanineCV replication cycle (21–26).

Although the replication of CanineCV has not been described, we can infer its replication process based on the well-documented replication mechanism of PCVs, especially PCV1 and PCV2, with the following steps (Figure 2): [1] The entry begins with the virus attaching to the host cell surface. This process is mediated by interactions between viral proteins and specific receptors on the host cell membrane. Entry primarily occurs through clathrin-mediated endocytosis, where the virus is engulfed into an endocytic vesicle and transported into the cell. [2] After entry into the cell, the viral capsid is uncoated, releasing the viral genome into the cytoplasm. The uncoating process involves the fusion of the endocytic vesicle with lysosomes, where the acidic pH facilitates the release of the single-stranded circular DNA from the protein capsid, which is transported to the cell nucleus, where replication occurs. [3] The replication of the virus genome occurs in the host cell nucleus. The Rep protein recognizes and binds to the origin of replication on the viral DNA. The Rep protein has endonuclease activity, which creates a nick in the DNA strand, producing a free 3′ OH end essential for new DNA strand synthesis. Using the 3′ OH end as a primer, the host DNA polymerase extends the DNA strand, synthesizing a new strand complementary to the original template strand. This results in the formation of a double-stranded replicative form (RF) DNA structure, which serves as a template for the synthesis of new viral single-stranded DNA through the rolling circle replication (RCR) mechanism. Replication is completed when the synthesis of the new DNA strand forms a full circle and meets the original 5′ end. The Rep protein makes another nick to release the new single-stranded DNA, which can be encapsulated into new viral particles. [4] The assembly of new viral particles occurs in the host cell nucleus. The capsid proteins (Cap), encoded by the ORF2 gene, are synthesized and transported to the nucleus, where they encapsulate the newly synthesized viral DNA. This assembly process involves forming complete viral capsids that enclose the viral DNA genome, creating new virions. [5] After assembly, the complete virions are transported out of the nucleus and accumulate in the cytoplasm before being released from the infected cell. Release can occur through cell lysis, where the host cell is destroyed, releasing virions into the extracellular environment. Alternatively, virions can be released through exocytosis, where vesicles containing virions fuse with the plasma membrane, releasing virions outside the cell without causing immediate host cell death. The replication process is carried out by cellular enzymes that are expressed during the S-phase of the host cell cycle (21–26).

There is still a lack of clarity regarding the physicochemical properties, replication process, and pathogenic characteristics of CanineCV. A study was conducted, rescuing a strain of canine circovirus in F81 cells using infectious clone plasmids, and it was discovered that the Rep protein produced by the viral packaging rescue process is associated with cytopathic effects. The Rep protein of CanineCV inhibited the activation of the type I Interferon (IFN-I) promoter, blocking the subsequent expression of interferon-stimulated genes (27).

Like observed in other circoviruses, a high evolutionary rate of 1.21 × 10^−3^ substitutions/site/year was described (66). This can be confirmed by the different genotypes that have been described since its first identification in 2012 (8, 17–19, 28, 29).

Phylogenetic analyses of the strains reported to date have been conducted using the complete genome sequences. These analyses also incorporate the nucleotide sequences or concatenated amino acids of the Rep and Cap proteins (24, 25, 66). Multiple efforts have been made to establish a classification system that helps understand virus origin and evolution. However, based on most recent articles where sequencing has been performed, the classification into six genotypes, i.e., CanineCV 1 to CanineCV 6, has been the most used and accepted (24, 25, 28, 30–33).

Phylogenetic analyses indicate that CanineCV likely originated from bat circovirus (BatACV). Maximum clade credibility (MCC) and maximum-likelihood (ML) trees constructed from ORF1 gene sequences suggest a close relationship between CanineCV and BatACV strains. This hypothesis is supported by the observation that circoviruses, including CanineCV, often undergo cross-species transmission, a major driver of their evolution. The genetic variations are often reflected in the virus's codon usage patterns, which have been influenced predominantly by natural selection rather than mutation pressure. This natural selection is a significant force shaping the codon usage bias (CUB) of CanineCV, enhancing its adaptability and survival in various hosts (16, 20, 33–36).

Codon adaptation index (CAI) and relative codon deoptimization index (RCDI) analyses have revealed that CanineCV exhibits the highest adaptability to red foxes, followed by domestic dogs and arctic foxes. This adaptability is attributed to the virus's ability to optimize its protein synthesis machinery to align with the host's codon usage preferences, thereby enhancing its replication efficiency and fitness. Interestingly, while CanineCV shows strong ties with wolves based on SiD analysis, the virus has developed the strongest adaptation to red foxes, indicating a complex interplay of host-specific adaptations driven by natural selection (34, 36).

## Epidemiology

Canine circovirus has been detected on every continent except Oceania (14–19).

**Figure 3:**
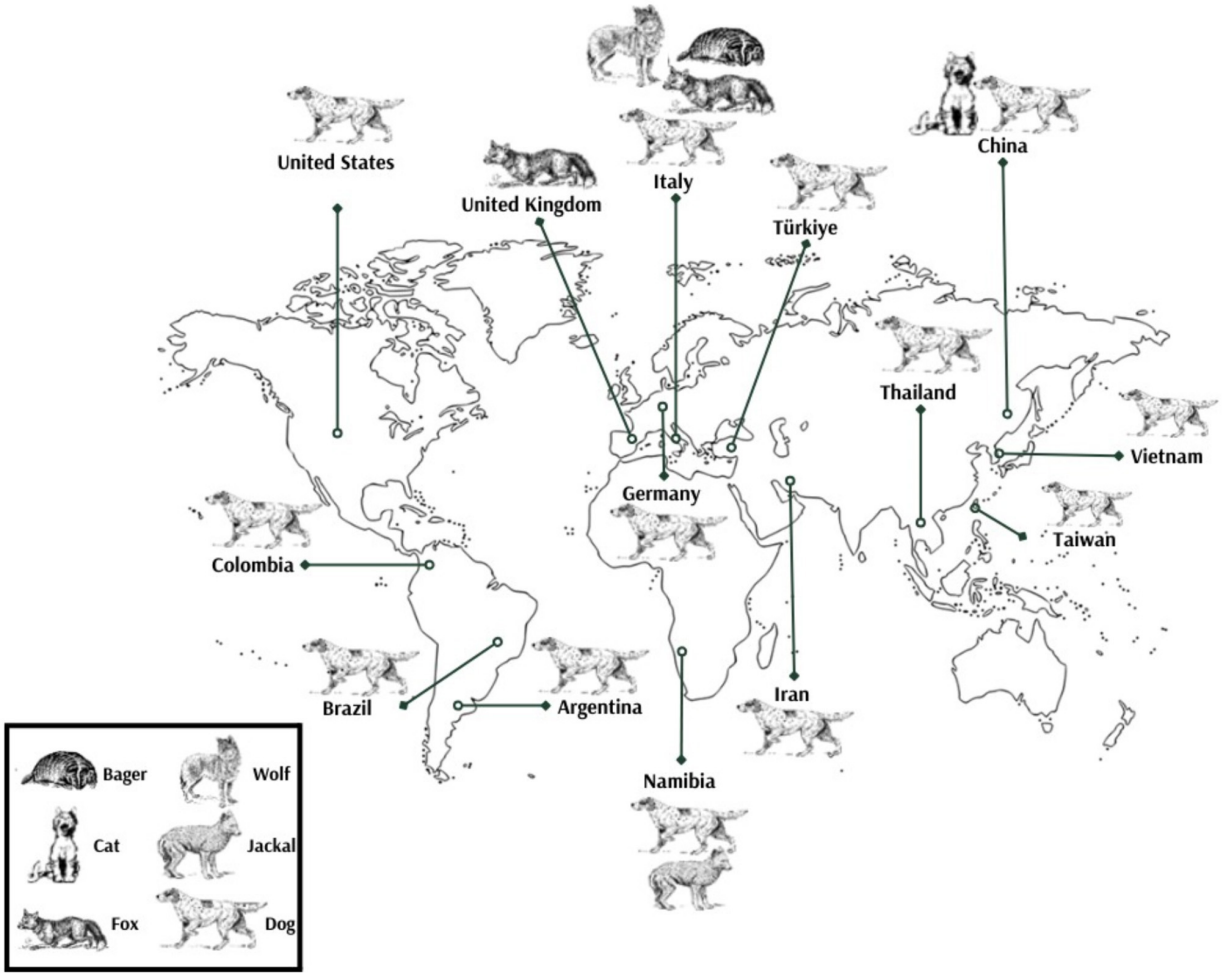
Global distribution of CanineCV detection across various species.

In the United States, the virus was first identified in 2012, followed by Italy in 2014, and the United Kingdom in 2015. Subsequent detections occurred in Taiwan (2016), Germany (2017), and Thailand (2017). Brazil reported its first case in 2018, with Argentina following in 2019. China, Turkey, and Colombia all recorded their initial detections in 2020. Vietnam identified the virus in 2020, Iran in 2022, and Namibia in 2023. These findings illustrate the widespread and chronological emergence of CanineCV across multiple continents, highlighting its global distribution [(8, 15, 16, 19, 20, 31, 35, 37, 38, 41–43, 57, 60, 67); Figure 3].

Retrospective studies have shown that CanineCV was present in Latin America as early as 2012 (43). In Europe, detection of the virus dates back to samples from 1995, indicating a longer and possibly more widespread historical presence of CanineCV in canine populations across different continents (68).

Canine circovirus has been identified in various host species, demonstrating its capacity for cross-species transmission and adaptability. Most detections have been reported in dogs, starting from 2012 [(8, 16, 19, 20, 25, 31, 37–43); Figure 3].

The prevalence of CanineCV in dogs varies widely from 3.6% to 28.0%, depending on the presence and severity of clinical signs. This range indicates that clinical manifestations play a significant role in the detection rates of CanineCV among domestic dogs (30, 44).

In wild carnivores, the prevalence of CanineCV shows considerable variation across different species. In foxes, the prevalence of CanineCV ranged from 0% to 4.3%. In badgers, the prevalence was 18%. In jackals, the prevalence was notably high at 43.7%, while wolves exhibited an even higher prevalence of 50% (15, 18, 30, 45, 46).

The classification into six genotypes, has revealed various geographic and host-specific distributions. CanineCV-1 has been detected in dogs primarily in China, USA, Colombia, Argentina, Italy, Germany, and Vietnam. Additionally, it has been found in wolves in Italy. CanineCV-2 has been found in dogs exclusively in China. CanineCV-3 has been detected in dogs in China, Vietnam, and Thailand. CanineCV-4 has been observed in both wolves and dogs in Italy, as well as in dogs in China, Germany, Argentina, and Colombia. CanineCV-5 has been found in Arctic foxes (*Vulpes lagopus*) and red foxes (*Vulpes vulpes*) in the Arctic, Norway, and the United Kingdom. Finally, CanineCV-6 has been detected in dogs in Iran [(8, 18, 19, 24, 29–33, 47); Table 1].

**Table 1:** Global distribution and host range of CanineCV genotypes.

**Genotype**	**Animal species**	**Countries**
CanineCV 1	Dogs	China; USA; Colombia; Argentina; Italy; Germany; Vietnam
	Wolve	Italy
CanineCV 2	Dogs	China
CanineCV 3	Dogs	China; Vietnam; Thailand
CanineCV 4	Wolve; dogs	Italy
	Dogs	China; Germany; Argentina; Colombia
CanineCV 5	*Vulpes lagopus*	Artic
	*Vulpes vulpes*	Norway; United Kingdom
CanineCV 6	Dogs	Iran

Canine circovirus has been detected in numerous tissue types, including the brain (dog and wolves), intestine (dog, wolves, and badgers), liver (dog), spleen (dog, wolves, fox, and badgers), lymph nodes (dog and jackals), and lungs (dog, wolves, and jackals). This extensive range of sample types demonstrates the virus's ability to infect and persist in different organs and tissues, contributing to its maintenance and spread within and between species [(8, 15, 28, 30, 45, 46); Table 2]. The detection of the virus in such a wide array of tissues highlights its versatility and pathogenic potential. Understanding the tissue tropism of the virus is crucial for developing effective strategies to control its spread and mitigate its impact on both domestic and wild animal populations. Further research is necessary to elucidate the mechanisms behind the virus's tissue-specific infection and its implications for disease progression and transmission.

**Table 2:** Detection of CanineCV in various host species and tissue type.

**Species**	**Year first detection**	**Samples**
Dog	2012	Feces, tissue^*^, nasal swab, and serum
Cat	2018	Nasal swab and serum
Fox	2010	Spleen
Wolves	2014	Tissue^#^
Badgers	2013	Spleen and intestine
Jackals	2021	Lymph node and lung

^*^Brain, intestine, liver, spleen, lymph nodes and lungs; ^#^Brain, lungs, spleen and intestine.

**Figure 4:**
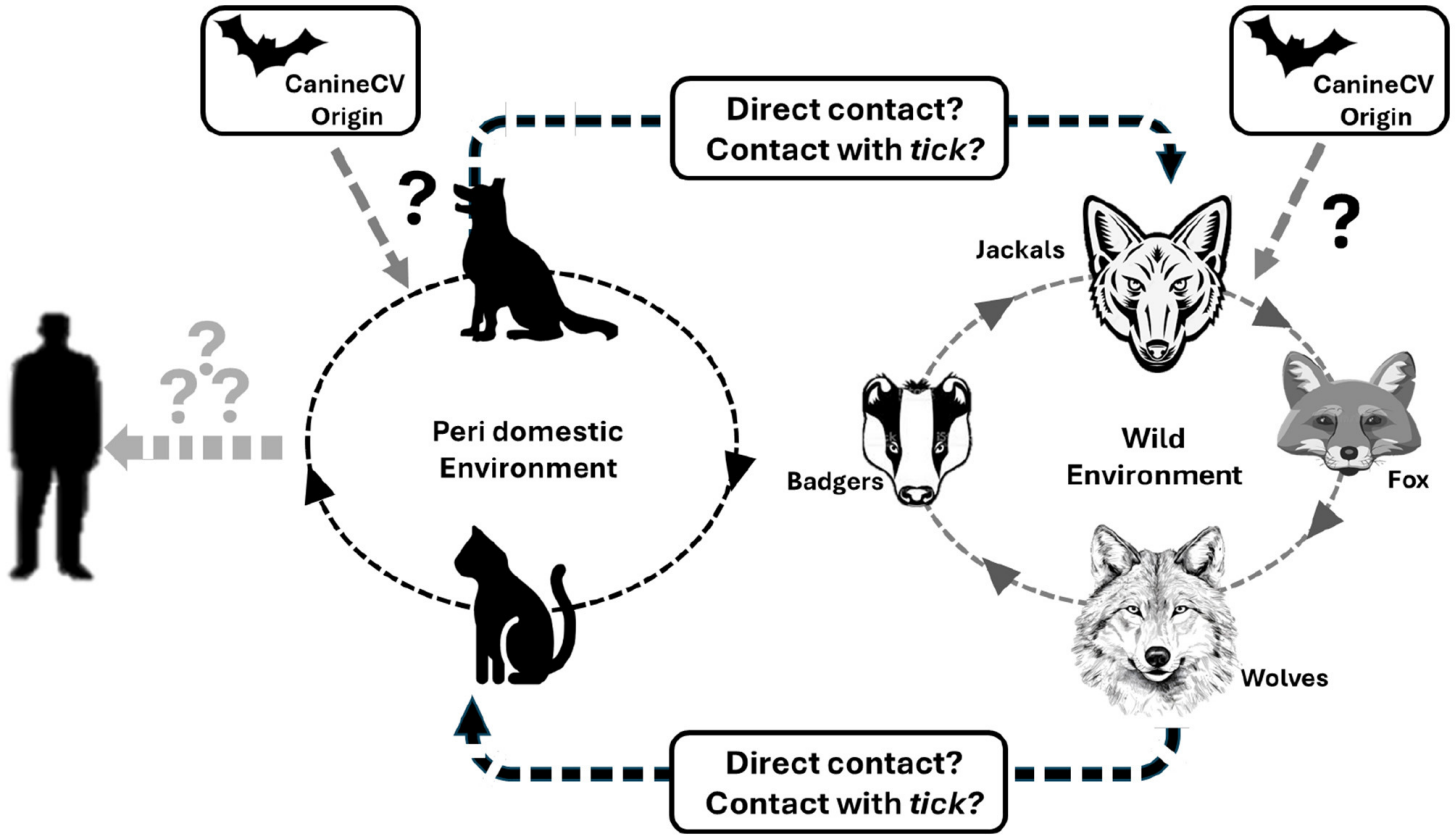
Schematic representation of potential occurrence CanineCV transmission route across different species is a potential occurrence based on the available literature. Indicates possibility of transmission that may have started in the peridomestic or wild environment indicates a remote possibility of transmission (9, 50).

Like *Porcine circovirus* 3 (PCV3), which infects swine, CanineCV originated from bat circovirus (BatACV). This ancestral virus may have adapted either directly to domestic dogs or through other intermediate hosts, allowing for cross-species transmission. Notably, PCV3 has been detected in ticks, and although CanineCV has not yet been described in ticks, this possibility should be considered. Figure 4 illustrates the potential transmission routes of CanineCV among different environments and species. It highlights direct contact and possible tick-mediated transmission as mechanisms through which the virus may spread among peri-domestic animals such as dogs and cats, and wild animals including foxes, badgers, jackals and wolves (9, 16, 33, 44, 48, 49). The figure underscores the complexity of CanineCV transmission dynamics and the need for further research to understand these interactions and their implications for viral maintenance and spread in diverse ecological setting (Figure 4).

The spillover events should be closely monitored due to the high mutation rate of CanineCV and recent socio-economic changes that have increased the proximity of companion animals to wild environments. Understanding these dynamics is crucial for developing effective strategies to control the spread of CanineCV and mitigate its impact on both domestic and wild animal populations (50).

The primary route of transmission for CanineCV is fecal-oral, affecting both domestic and wild animals. Viral loads in feces have been detected at 1.8 × 10^3^ copies of target DNA/μL of extracted DNA in dogs and 8.94 × 10^4^ copies in foxes. Notably, animals without clinical signs also shed high viral loads in their feces, as indicated by a low cycle threshold (*Ct*) value of 20.7 (31, 32, 45). It must be considered that the high amount of viral particles is an important factor for viral spread in animal populations.

Special attention in the epidemiological chain should be given to the fact that some animals, such as dogs, cat, foxes, jackals, and wolves, may be infected without showing clinical signs, as they can still disseminate the virus. However, it should be considered that, like swine, the viral load shed by asymptomatic animals is lower. This highlights the importance of monitoring both symptomatic and asymptomatic carriers to effectively control the spread of CanineCV in various animal populations (15, 28, 30, 51, 52).

While the fecal-oral route is the primary mode of transmission for CanineCV, the potential for respiratory transmission also warrants attention. Although few studies have investigated this route, the virus has been detected in respiratory samples, indicating that respiratory transmission could be a significant pathway for the spread of CanineCV (28, 29). Further research is necessary to understand the extent and implications of respiratory transmission in both domestic and wild animal populations.

The prevalence of CanineCV varied significantly by age group. Among dogs aged 0–1 years, the prevalence ranged from 17.5% to 43.1%, indicating a higher susceptibility in this age group. For dogs aged 1–8 years, the prevalence ranged from 9.6% to 43.1%. In dogs older than 8 years, the prevalence was consistently reported at 0% to 13.8%. Additionally, for dogs with unreported ages, the prevalence was noted to be 13.2%. These findings highlight the significant differences in CanineCV prevalence across age groups, with the highest rates observed in the youngest dogs (14, 15, 29–32, 38, 42, 44, 53).

The prevalence of CanineCV in wolves showed significant variation across different age groups. The overall prevalence was 47.8%, with 43.5% of infected wolves being puppies (<12 months old), 30.4% being sub-adults (13–24 months), and 26.1% being adults (older than 24 months). Specifically, the prevalence was highest in puppies at 50% (5/10), followed by sub-adults at 42.9% (3/7), and adults also at 50% (3/6). These findings highlight that CanineCV affects wolves across all age groups, with a notable prevalence in both the youngest and oldest age categories, differently from what is seen in dogs (15, 30, 46).

Sex-based analysis of CanineCV infection rates revealed a higher prevalence in female dogs compared to male dogs, although this difference was not statistically significant. The prevalence in female dogs ranged from 57.1% to 67.6%, while in male dogs it ranged from 32.4% to 42.9% (31, 53).

## Clinical signs

The virus's ability to infect and persist in multiple tissues not only aids in its dissemination but also in its pathogenicity, contributing to a range of clinical manifestations in infected hosts. In CanineCV infected animals, most clinical signs are related to the digestive system but are also associated with the respiratory and nervous systems.

### Cats and dogs

The prevalence of CanineCV among symptomatic and asymptomatic animals shows significant variability. On average, 10.6% of asymptomatic animals are infected, with a range between 6.9% and 28.5%. Among symptomatic animals, the average prevalence is higher, at 20.3%, with a range between 6% and 32.8%. In cats, a similar pattern is observed, with a higher prevalence of CanineCV in symptomatic animals (3.6%) compared to asymptomatic ones (1.1%). These findings show that CanineCV infection is present in both symptomatic and asymptomatic populations, with a notably higher prevalence in those showing clinical signs (14, 15, 18, 24, 28, 29, 31, 32, 38, 42, 53, 54).

**Figure 5:**
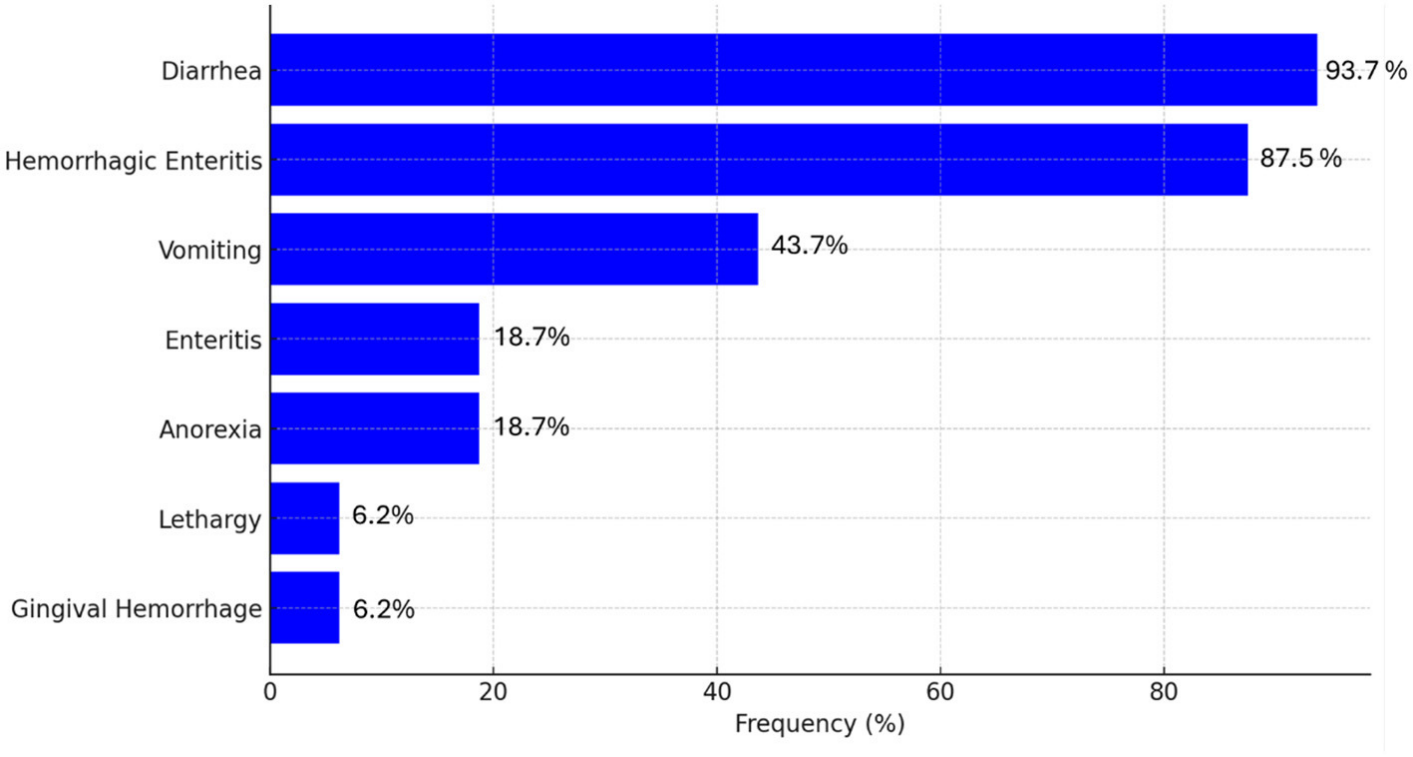
Overall prevalence (%) of clinical signs in dogs infected with CanineCV 3 based on consulted literature.

Considering 16 articles describing clinical signs, a ranking of the most common clinical signs associated with CanineCV, as shown in Figure 5, indicates that enteric disturbances are the most frequently observed. Diarrhea was the most prevalent symptom, observed in 93.7% of cases, followed by hemorrhagic enteritis, which occurred in 87.5% of cases. Vomiting was documented in 43.7% of the cases, while anorexia and enteritis were less frequent, each with a prevalence of 18.7%. Lethargy and gingival hemorrhage were the least common symptoms, each observed in 6.2% of cases [(14, 20, 24, 28, 31, 32, 38, 42, 51, 53–58); Figure 5].

In addition to these more common signs reported in studies, there are studies associating CanineCV with respiratory (28, 59) and nervous signs (58) and lymphadenitis (31).

As observed with CanineCV involvement digestive systems clinical signs, the prevalence of the virus in animals with respiratory illness is also higher, as demonstrated in this study linking respiratory diseases to CanineCV. The overall occurrence of CanineCV infection was 8.95% (17/190), with 2.6% (2/76) in the healthy group and 13.2% (15/114) in the respiratory illness group and several factors can influence its prevalence. Age-wise, juniors (<1.5 years) exhibited the highest positivity rate at 17.5%, compared to adults (1.5–6 years) at 10.5% and seniors (≥6 years) at 1.3%. Sex-wise, females showed a higher positivity rate at 10.5% compared to males at 5.5% (29).

There are few studies that describe histopathological lesions, most of which are associated with enteric disorders. In a case report from Connecticut, United States, microscopic examination revealed major lesions in the gastrointestinal tract confined to the small intestine. These included random crypt cell necrosis and focal hemorrhage in the lamina propria and submucosa. Vascular changes comprised endothelial cell swelling and sloughing, leading to endothelial disruption. Vasculitis was noted in small arterioles of the basal mucosa and submucosa, rarely accompanied by thrombi. Hyaline degeneration and fibrinoid necrosis of small vessels were occasionally observed (32, 58, 59).

Histologically, CanineCV-positive dogs with respiratory illness exhibited varying severities of generalized hemorrhagic pyogranulomatous pneumonia, multifocal hemorrhagic pneumonia, and severe diffuse suppurative, hemorrhagic, and necrotic bronchiolitis and alveolitis (29).

Other organs such as the brain, meninges, myocardium, lung, liver, and kidney exhibited severe focal vasculitis with mononuclear cell inflammation. The spleen and lymph nodes showed significant lymphocyte necrosis and hemorrhage. Histological analysis revealed hyaline degeneration, fibrinoid necrosis of small vessels, and marked sinus histiocytosis in lymph nodes (58–60).

As observed in other animal species, such as swine, PCVs co-infected with other viruses or bacteria have been demonstrated to enhance PCV2 and PCV3 replication in target tissues. This co-infection increases the severity of induced lesions and exacerbates the clinical course of the disease. The presence of concurrent infections significantly impacts the pathogenesis, leading to more severe clinical manifestations and challenging disease management (9, 52). Studies have shown that CanineCV can co-infect with various other pathogens, the most common being canine parvovirus (CPV), canine adenovirus (CAdV), coronavirus (CCoV), and distemper virus (CDV), generally resulting in severe clinical signs.

In double co-infections, co-infection with CPV varied between 18.7% and 57.7% (54, 61), while CPV2 co-infection rates were 15.6% (15/96) and 16.6% (33, 41). CCoV was found in 12.67% of cases (54). The frequencies of triple co-infections involving CPV, CCoV, and CanineCV ranged from 3.2% to 9.8%. A retrospective study analyzing 95 samples of enteritis caused by parvovirus found that 8 (8.9%) were positive for CanineCV (30).

In addition to viral co-infections, double and triple infections with others agents were also related with enterotoxigenic *Escherichia coli*, Shiga toxin-producing *Escherichia coli*, Salmonella, *Cryptosporidium* spp., *C. perfringens* α toxin, *Giardia* spp., *Campylobacter jejuni*, and *Campylobacter coli* were also described, although their frequencies were not reported (59, 60, 62).

Co-infection was also observed in animals in studies investigating the association between CanineCV and respiratory diseases. Among CanineCV-positive dogs, nine (52.94%) were co-detected with other pathogens: canine herpesvirus 1 (CaHV-1; *n* = 2), canine distemper virus (CDV; *n* = 2), canine respiratory coronavirus (CRCoV; *n* = 2), canine parainfluenza virus (CPIV; *n* = 1), canine adenovirus type 2 (CAdV-2; *n* = 1), and triple-detected with CaHV-1 and CRCoV [*n* = 1; (29)].

The association between viral load and disease severity has been described in PCV2 infections in swine, where a threshold viral load correlates with clinical signs. There for, 10^7^ or greater PCV2 genomic copies per milliliter of serum were associated with severe PCV2-associated disease (PCVAD), and poor prognosis. Consequently, PCR results are reported as negative, positive with no PCVAD (<10^6^ PCV2 DNA copies), positive with PCVAD suspect (10^6^ PCV2 DNA copies), or positive with PCVAD [10^7^ PCV2 DNA copies or greater; (52)].

In CanineCV infections, viral loads varied between 3.57 × 10^1^ and 8.37 × 10^8^ (30, 54). Another method used to determine viral load in studies was Cycle Threshold (*Ct*) values, which are inversely proportional to viral load; lower *Ct* values indicate higher viral loads. *Ct* values ranged from <13 to 30 (18, 32). Although there is no standardization of viral load and disease severity for CanineCV, *Ct* < 13 in intestinal samples of three dogs from a study were associated with severe clinical signs such as anorexia, vomiting, and severe bloody diarrhea during outbreaks in a Papillon breeding colony in Michigan in March 2013 and February 2014 (32). The association between viral load and disease severity was also observed, with CanineCV loads generally low, ranging from 3.57 × 10^1^ to 8.37 × 10^8^ (mean of 1.03 × 10^3^) and from 8.60 × 10^1^ to 5.38 × 10^5^ viral DNA copies/μL (mean of 2.45 × 10^2^) for clinical cases and control animals, respectively (54).

### Wild animals

Different studies have confirmed the presence of CanineCV in wild carnivores, including wolves, foxes, badgers, and jackals, with wolves being the most studied. These studies are concentrated in Italy and Africa. In wolves, the prevalence of CanineCV varies between 26.4% and 50% of the animals tested, using tissue samples such as intestine and spleen. The overall median quantity of CanineCV DNA was 6.8 × 10^2^ copies of the target DNA per microliter of template [range: 8.2 × 10°−3.7 × 10^7^; (18, 30, 46)].

In wolves, co-infection with CanineCV and other pathogens has been reported in only two studies. One study identified co-infection in 47.8% (11/23) of wolves, with 72.7% (8/11) involving Carnivore protoparvovirus 1 and CanineCV. Additionally, 18.2% (2/11) tested positive for three viruses: one case with Carnivore protoparvovirus 1, CAdV-2, and CanineCV, and another with Carnivore protoparvovirus 1, CAdV-1, and CanineCV (30). Another study found that CanineCV was detected alongside CDV in 77.8% (7/9), CPV-2 in 44.4% (4/9), and *Trichinella britovi* in 22.2% (2/9) of the cases. Co-infection with two or three agents, in addition to CanineCV, was observed in 22.2% (2/9; CPV-2 + CDV), 11.1% (1/9; CDV + *Trichinella britovi*), and 22.2% (2/9; CPV-2 + CDV + *Trichinella britovi*) (46).

In foxes, the prevalence of CanineCV varied from 0% (0/232) to 4.3% (5/115) (17, 66). The viral load ranged from 1.96 to 8.94 × 10^4^ copies of DNA/mL of tissue homogenate (pool of organs or spleen), reflecting variations in viral replication or the stage of infection at the time of sampling. The only CanineCV-positive animal that did not die from trauma (1/5) presented neurological symptoms (46).

The only study involving jackals was conducted on samples (lung and lymph node) collected during predator control operations in 2021, and the prevalence was 18%. Therefore, no clinical signs or diseases were associated with CanineCV infection in this study. The prevalence of CanineCV in jackals was 43.75% [14/32; (15)].

Recently, a novel circovirus was identified in Iberian lynxes (*Lynx pardinus*), one of the most endangered feline species in the world and a symbol of wildlife conservation in Europe. Study conducted by Castro-Scholten et al. (63) identified the Iberian lynx-associated circovirus-1 (ILCV-1) in 57.8% of spleen samples analyzed, collected from both wild and captive populations. The high positivity rate observed suggests a systemic infection that may have significant implications for the immunological and overall health of this species. Iberian lynxes, which already face substantial challenges due to habitat loss, prey scarcity, and infectious diseases such as bovine tuberculosis and feline leukemia virus, now confront a new potential pathogen that could further complicate conservation efforts (63). The discovery of ILCV-1 highlights the urgent need for additional studies to better understand the epidemiology, clinical impact, and potential transmission mechanisms of this virus, as well as to evaluate management strategies to mitigate the risks associated with its circulation in already vulnerable populations. This identification also broadens our understanding of viral diversity in large felines and underscores the importance of systematic virological investigations in endangered species.

## Diagnostic

One of the most widely used techniques to detect the CanineCV genome is the Polymerase Chain Reaction [PCR; (14, 20, 24)] particularly real-time quantitative PCR (qPCR). PCR has also been employed for sequencing purposes (14, 15, 25, 29–31, 41, 45, 53, 57, 60).

The systems utilized for qPCR include SYBR Green (18.5%) and Taqman (81.5%). Several studies have utilized qPCR qualitatively due to its ability to be up to 1,000 times more sensitive than traditional PCR, while others have used qPCR to quantify CanineCV DNA in various types of samples (15, 17, 19, 31, 33, 38, 46, 48, 55, 64).

The advantage of qPCR lies in its ability to establish the absolute quantification of viral nucleic acid. As indicated above, the quantification of CanineCV in canine tissues (18, 29, 30, 32, 57), wolves (30), and foxes (45) allows for determining the target tissues for replication that may be related to the virus's pathogenesis. Additionally, the quantification of CanineCV in feces (32, 43, 54, 68) and nasal secretions (29) lays a significant role in understanding viral dissemination (Table 3).

**Table 3:** Summary of methods and sample types used for CanineCV detection across different species and regions.

**Method**	**Country**	**Species**	**Sample**	**References**
PCRq^*^	Africa	Dog and jackals	Lung and lymph node	(15)
PCRq^*^	Africa	Dog	Serum	(18)
PCRq^*^	Brazil	Dog	Feces	(56)
PCRq^**^	Brazil	Dog	Lung, liver, and spleen	(64)
PCR	Brazil	Dog	Feces and fecal swab	(14)
PCRq	China	Dog	Feces	(33)
PCR^****^	China	Dog and cats	Fecal, nasal swabs, and serum	(28)
PCRq^*^	China	Dog	Blood samples	(59)
PCRq^**^	Colombia	Dog	Feces	(41)
PCRq^**^	Colombia	Dog	Feces	(25)
PCRq^*^	Germany	Dog	Feces	(55)
PCRq^*^	Iran	Dog	Rectal swabs	(31)
PCRq^*^	Iran	Dog	Feces	(53)
PCRq^*^	Italy	Dog	Liver and intestine	(57)
PCRq^*^	Italy	Dog, wolfs, foxes, and badgers	Tissue^###^	(46)
PCRq^*^	Italy	Dog	Feces and/or rectal swabs	(54)
SYBR Green-based qPCR^*****^	Italy	Wolfs	Tongue, intestine, and spleen	(30)
SYBR Green-based qPCR^*****^	Italy	Foxes	Pools of organs	(45)
SYBR Green-based qPCR^*****^	Italy	Dog	Faces or intestine	(30)
PCRq^*^	Italy	Wolfs, foxes, and badgers	Spleen and intestine	(18)
SYBR Green-based qPCR^*****^	Taiwan	Dog	Rectal swabs or feces	(38)
PCR/ISH	Thailand	Dog	Nasal, oral swabs, and tissue samples^#^	(20)
SYBR Green-based qPCR^*****^/ISH	Thailand	Dog	Nasal swab and lung	(29)
PCRq/ISH	USA	Dog	Feces, serum, and tissue^##^	(32)
PCRq^*^	USA	Dog	Intestine, liver, and spleen	(58)
PCRq Rep and Cap/ISH	USA	Dog	Blood, feces, and tissue	(60)
PCRq^***^	Vietnam	Dog	Fecal swabs	(19)

^*^PCRq by Li et al. (60); ^**^PCR by Kotsias et al. (16); ^***^PCRq by Piewbang et al. (20); ^****^PCR by Hao et al. (27); ^*****^PCRq by De Arcangeli et al. (82); ^#^Brain, lung, liver, kidneys, tonsil, and tracheobronchial lymph nodes; ^##^Lung, liver, spleen, and intestine; ^###^Spleen, tonsil, lymph nodes, liver, intestine, lung, kidney, brain.

The primer sets used in both PCR and qPCR reactions target the cap gene (60) the Rep gene (29, 60), and intergenic region (65). The Rep region and intergenic region, due to their lower mutation rates compared to the cap region, should be considered in diagnostics as they enhance the detection of viruses that may have undergone mutations.

Although several PCR techniques are currently in use, considerable research is still being conducted to standardize and validate these methods to optimize the diagnosis of CanineCV.

Hao et al. (27) developed multiplex PCR (mPCR) method demonstrated superior results compared to traditional PCR techniques, offering simultaneous detection of multiple canine viruses, including canine adenovirus type 2 (CAV-2), canine influenza virus (CIV), CD, CPIV, CanineCV, CCoV, and CPV, with high sensitivity and specificity. The mPCR method's detection limit was established at 1 × 10^4^ viral copies for both respiratory and enteric viruses, significantly enhancing diagnostic accuracy in clinical samples. The ability to detect up to seven different viruses in a single reaction not only streamlines the diagnostic process but also improves the reliability of detecting co-infections. This method, therefore, presents a valuable tool for comprehensive epidemiological surveillance and the rapid, precise diagnosis of canine viral infections.

Still with the aim of diagnosing agents involved in CanineCV co-infections, Wang et al. (36) developed a duplex SYBR Green I-based real-time PCR assay developed for the simultaneous detection of CanineCV and CaAstV demonstrated high sensitivity and specificity. The assay's detection limits were 9.25 × 10^1^ copies/μL for CanineCV and 6.15 × 10^1^ copies/μL for CaAstV, making it significantly more sensitive than traditional PCR methods. The duplex PCR also showed no cross-reactivity with other common canine viruses, such as CPV, CCoV, CDV, and canine kobuvirus (CaKoV), underscoring its specificity. Additionally, the reproducibility of the assay was confirmed through low intra- and inter-assay variation. This method offers a rapid, reliable, and cost-effective tool for detecting co-infections in clinical samples, significantly improving the accuracy of diagnosis in cases where CanineCV and CaAstV are suspected.

Chip digital PCR (cdPCR) is a cutting-edge PCR method that involves encapsulating nanoliter-sized volumes of liquid in high-throughput microcells or microchannels for PCR amplification, followed by direct interpretation of fluorescence signals. This technique allows for the absolute quantification of nucleic acids without the need for external standards, calibration curves, or Ct values. cdPCR excels in precisely detecting and measuring even very small amounts of DNA, making it especially useful for samples with low DNA concentrations or those that contain inhibitors that could interfere with traditional PCR methods. The technique is also known for its high sensitivity and specificity, significantly minimizing the chances of false positives or negatives. This method was used for the detection of CanineCV and exhibited a detection limit of 6.62 copies/μL, making it ~10 times more sensitive than qPCR, which had a detection limit of 6.62 × 10^1^ copies/μL. This increased sensitivity allows for more accurate detection, especially in samples with low viral loads. Furthermore, the cdPCR method showed excellent specificity, with no cross-reactivity observed with other common canine viruses and demonstrated high repeatability with low intra-assay and inter-assay coefficients of variation (36).

Another widely used technique for viral detection is *in situ* hybridization (ISH), which labels viral DNA within tissue samples, enabling precise localization of the virus. This technique is crucial for identifying target tissues and understanding the lesions associated with viral infections. By determining the exact location of the virus in the tissue, ISH provides valuable insights into the pathogenesis of the infection and its impact on specific tissues. In this technique, the Rep gene has been used as the target for detecting CanineCV DNA within various tissues of infected dogs (29, 32, 59).

*In situ* hybridization was particularly effective in identifying the presence of viral nucleic acid within specific lymphoid tissues, such as the spleen, mesenteric lymph nodes, and Peyer's patches. The ISH method produced strong positive signals in these lymphoid tissues, especially within epithelioid macrophages located in regions of granulomatous inflammation (32, 59). Additionally, ISH located CanineCV DNA within the pulmonary tissues, notably within the alveolar lining cells, endothelial cells of capillary blood vessels, and lymphoid cells within the follicles of the tracheobronchial lymph nodes. The technique demonstrated high sensitivity, successfully detecting viral DNA within the nuclei and cytoplasm of histiocytes and macrophages. This precise localization of the virus in both lymphoid and pulmonary tissues highlights the direct association between CanineCV and the pathological lesions observed in these areas (69). By providing detailed insights into the specific tissues affected and the cellular localization of the virus, ISH proves to be a critical tool in understanding the pathogenesis of CanineCV infections.

## One Health

The One Health concept, defined as the collaborative effort of multiple disciplines working locally, nationally, and globally to attain optimal health for people, animals, and the environment, has gained significant recognition. This approach acknowledges the interconnectedness of human, domestic animal, and wildlife health within the broader context of ecosystem health. By providing a holistic framework, One Health facilitates the development of comprehensive solutions to global health challenges. The emergence of infectious diseases, whether novel or known, exemplifies the dynamic interplay between pathogens, hosts, and their environments, highlighting the necessity of an integrated approach to health (70–72).

The proximity between wild and domestic hosts plays a crucial role in the transmission of viruses. As human populations expand and urbanize, the interactions between humans, domestic animals, and wildlife increase, heightening the risk of pathogen transmission and the emergence of novel disease outbreaks. Factors such as wildlife trade and the introduction of domestic species decrease the geographical and behavioral separation between donor and recipient hosts, promoting viral emergence. These interactions create opportunities for cross-host exposures, a critical step in the transference to new hosts, and facilitate the establishment of epidemics by enabling sufficient contact for virus transfer and adaptation (71, 74–76).

RNA and single-stranded DNA (ssDNA) viruses exhibit high mutation and nucleotide substitution rates, allowing rapid evolution and adaptation to new environments. This high variability, coupled with error-prone replication and the lack of a proofreading mechanism, enhances their ability to infect new hosts. For instance, RNA viruses have rapid replication, short generation times, and large populations, which increase the likelihood of adaptation to new hosts. In contrast, most DNA viruses are less variable, often showing virus-host co-speciation. However, ssDNA viruses like *Circoviridae* can exhibit mutation rates like RNA viruses, suggesting their potential for rapid evolution and cross-species transmission. These rapid evolutionary capabilities are particularly concerning when considering the increased interactions between humans, domestic animals, and wildlife (77–80).

The behavior of CanineCV and PCVs highlights the complexities of viral adaptation and cross-species transmission. PCV3, a member of the *Circoviridae* family, has been shown to infect multiple hosts, with a high possibility of infecting baboons, demonstrating its capability for trans-species transmission. CanineCV, with its high mutation rate, can adapt to various hosts, like the behavior observed in PCV (9, 24, 25).

Adaptation to interhost transmission by droplet spread, and fecal-oral transmission, which occur with the CanineCV, represent different adaptational challenges due to host differences and variation in environmental exposure, therefore the capacity of the virus in the environment is very important (75). There is no study that shows the viability of CanineCV in the environment, but there is with PCV. PCV2 were detected in wastewater from manure treatment systems consisting of an equalization tank, a settling tank, an anaerobic reactor, an aerobic reactor, and a secondary settling tank, showing its stability in the environment (81). The survival of the virus in the environment is a crucial factor in the spread of the virus and increases the possibility of the virus contacting new hosts.

Another important factor that must be considered is the dog meat feeding habits in some countries. Additionally, serum from these animals may has a high viral load, with a *Ct* ranging from 28 to 35, making a possible human route of infection, not only through ingestion but also during handling of the animals during slaughter. *t* should also be noted that depending on the moment of infection, the amount of virus may be even greater, with a *Ct* range of 13–30 (18, 32).

The detection of CanineCV across these various host species highlights the virus's adaptability and potential for cross-species transmission. Further research is necessary to understand the mechanisms behind this adaptability and the implications for disease management in both domestic and wild animal populations.

## Conclusion

In conclusion, CanineCV represents a significant emerging pathogen with the ability to infect various species, including domestic dogs, wild carnivores, and potentially other hosts. The high genetic variability and adaptability of CanineCV, as evidenced by its widespread detection across different regions and host species, underscore the importance of continued surveillance and research. Diagnostic advancements, including the use of techniques such as qPCR, ISH, and multiplex PCR, have significantly enhanced our ability to detect and quantify the virus, thereby improving our understanding of its epidemiology and pathogenicity. These tools, combined with detailed phylogenetic analyses, are crucial in monitoring the virus's evolution and in developing strategies to mitigate its impact on animal health. The observed associations between CanineCV infections and co-infections with other pathogens highlight the complex interplay between the virus and host immune responses, which can exacerbate disease severity.

Therefore, ongoing research into the virus's transmission dynamics, tissue tropism, and interactions with co-infecting agents is essential for developing effective control measures and understanding the broader implications of CanineCV infections for both domestic and wild animal populations.
